# Sex and Gender Differences in Face and Upper Extremity Allotransplantation: A Narrative Review of Implications and Impact

**DOI:** 10.7759/cureus.77938

**Published:** 2025-01-24

**Authors:** Zeynep Demir, Naga Anvesh Kodali, Omer Faruk Dirican, Bedreddin Sazoglu, Ramu Janarthanan, Yalcin Kulahci, Fatih Zor, Vijay S Gorantla

**Affiliations:** 1 Surgery, Wake Forest School of Medicine, Winston-Salem, USA; 2 Plastic and Reconstructive Surgery, Amrita Institute of Medical Sciences and Research Centre, Amrita Vishwa Vidyapeetham, Kochi, IND; 3 Plastic and Reconstructive Surgery, Indiana University School of Medicine, Indianapolis, USA

**Keywords:** face transplantation, female, gender differences, hand transplantation, vascularized composite allotransplantation (vca)

## Abstract

Sex and gender differences play a significant role in vascularized composite allotransplantation (VCA), impacting both surgical outcomes and postoperative care. While sex refers to biological distinctions and gender encompasses life experiences and behaviors, both factors are closely interrelated in transplantation. Biological sex differences, such as immune responses, hormonal fluctuations, and anatomical features influence graft adaptation and healing, while both sex and gender-specific factors affect psychological and functional recovery. Studies indicate that men have higher mortality and reoperation rates, whereas women are more susceptible to adverse effects from immunosuppressive therapies. Unlike solid organ transplantation (SOT), which is primarily lifesaving, VCA focuses on life-enhancing outcomes, requiring careful attention to patients' social, psychological, and functional well-being. Lifelong immunosuppression in VCA carries risks, including infections and cancer, complicating patient management. Psychological readiness is crucial for candidate selection and long-term success, with women often expressing greater concerns about outcomes. Although no significant gender differences in functional recovery have been observed, individualized rehabilitation plans based on anatomical and physiological differences remain essential for optimal recovery.

## Introduction and background

Introduction

Since the first successful hand transplant in 1998 [1] by a team in Lyon, France, vascularized composite allotransplantation (VCA) has advanced significantly, enabling the transplantation of complex anatomical structures such as the larynx, tongue, and the entire face. The first human face transplant also performed in Lyon in 2005 [2], marked another milestone in this field. Despite the ethical, surgical, and patient follow-up challenges associated with VCA, these historic achievements have established it as one of the most effective reconstruction methods for patients with complex soft tissue and bone defects. Over the past three decades, VCA has become a vital component of reconstructive transplantation [3,4].

Despite the significant anatomical and physiological differences between sex and gender-specific organs, their role in VCA outcomes has not been well studied in the literature. While sex refers to biological differences, gender encompasses life experiences and behaviors. These concepts are deeply interconnected, as biology can influence behaviors and experiences, while life experiences can shape biological responses. Systematically addressing sex and gender differences in VCA outcomes is challenging due to the unique nature of each procedure. However, significant sex-based disparities have been identified in surgical outcomes. For instance, a 2018-2019 study by Jochum et al. involving 367,887 patients revealed that men had a 16% higher mortality rate and were 21% more likely to require reoperation within 90 days compared to women, even after adjusting for age, comorbidities, and surgical procedures [5]. These findings underscore the critical need to consider sex and gender as key variables in VCA research and practice [5].

However, after surgery, it becomes more predictable that sex and gender differences can influence the adaptation and healing of transplanted tissue, as well as the response to immunosuppressive therapies. For example, in a study involving 23,296 individuals (8,838 female) examining the side effects of chemotherapy and immunotherapy, women were found to have a nearly 50% higher risk of experiencing serious adverse effects compared to men [6]. This suggests that sex and gender differences may play a significant role in post-surgical recovery and treatment response, particularly in VCA.

## Review

Methods

This narrative review aims to explore and synthesize existing literature on sex and gender differences in face and upper extremity allotransplantation, focusing on physiological, immunological, and psychosocial aspects. A comprehensive literature search was conducted using electronic databases, including PubMed and Embase, covering the period from 1998 to 2024. The selected titles focused on topics including "Gender-Specific Differences in Transplant Outcomes," "Immunological Differences between Female and Male Transplantation," and "Psychological Differences between Female and Male Transplant Recipients." The search covered studies covering gender-specific information in VCA. We searched for the keywords "VCA", "Female", "Gender Differences", "Face Transplantation", "Hand Transplantation". This search yielded 202 articles related to face transplantation and 101 articles concerning hand transplantation. Data extracted from each eligible study included study design, sample size, patient demographics (specifically sex and gender), type of allotransplantation, measured outcomes, and any reported sex- or gender-based differences.

In this narrative review, we carefully assessed the quality and validity of studies by looking at their design, sample size, and how well they analyzed sex or gender differences. To minimize selection bias, we incorporated a diverse range of studies. However, the limited number of publications specifically addressing sex and gender differences in this area presents a limitation and affects the generalizability of our findings.

Citation management was handled using EndNote v21.5 (Clarivate Analytics, London, UK).

General differences between solid organ transplantation (SOT) and VCA

SOT focuses on maintaining the general health condition of the patients, often requiring healthcare professionals to make immediate decisions when there is any donor opportunity. In contrast, VCA addresses the integrity of the body, as well as social and psychological aspects, requiring both VCA candidates and healthcare professionals to take more time for decision-making. This distinction arises because solid organ transplantation is aimed at lifesaving [7], while VCA is focused on life-enhancing. Although both procedures require immunosuppression [8], one of the significant challenges in VCA procedures is ensuring that the recipient never develops tolerance induction despite lifelong immunosuppression therapy.

Navigating the future of VCA requires a delicate balance between the profound benefits it offers and the inherent risks and challenges it poses [9]. Addressing these medical, ethical, and social complexities is essential to advancing the field and ensuring that patients can safely experience the full potential of these transformative procedures.

Female recipients in hand and face transplantation

Sex compatibility between donor and recipient remains a significant and rate-limiting factor in both hand and face transplantation [10]. According to surveys of donor willingness and data from a five-year New England Organ Bank study, only an estimated 20% of solid organ donors are eligible for VCA [11]. Nearly half of VCA candidates wait more than a year, and many more than three years [12]. A 2020 survey of 860 individuals in the U.S. found that 20.8% were likely to become upper limb donors, with two-thirds of potential donors being female (66.7%) [13].

Published studies to date confirm that a total of nine (19.6%) female patients out of 48 patients underwent face transplantation (with one male and one female patient receiving two face transplants each) [14,15]. Among them, 39 patients are still alive (eight of them female cases) whilst, seven patients have died (mortality rate of 15.2%). The 36 out of 48 (75%) face transplant cases developed an acute rejection (AR) while only seven out of the total of 48 face transplant cases developed chronic rejection (CR) (14.6%). Table 1 summarizes face transplant cases in female patients performed until 2024 [1, 16-32].

**Table 1:** Female face transplantation until 2024. AR: acute rejection, CR: chronic rejection.

Surgical team	Date	Location	Patient age	Indication	Functions impaired	Anatomic defect and face allograft outcome	Rejection	Psychosocial
Devauchelle et al. [1]	November of 2005	Amiens, France	38 y	Animal attack	Labial competence, speech	Partial defect, died in 2016 due to malignancy	AR: Yes, CR: In 2014 and 2015	At 4 months: The patient returned to normal social life and accepted a new face, At 18 months: Not afraid to walk in the street and meet friends at a party
Siemionow et al. [19]	December of 2008	Cleveland, Ohio	45 y	Ballistic trauma	Eating/ drinking, ocular and periocular competence, speech, oro-myofunctional competence, facial expression	Partial defect, died in 2020 due to infection	AR: Yes, CR: No	At 11 months: Representing societal reintegration, At 3rd year: Appearance self-rating from 3/10–7/10, anxiety about body image
Pomahac et al. [21]	May of 2011	Boston, Massachusetts	57 y	Animal attack	Eyelid closure, speech	Full face defect, alive	AR: Yes, CR: No	At 24 months: Fluctuation in health status, consistently high self-esteem
Ozmen et al. [22]	March of 2012	Ankara, Turkey	20 y	Ballistic trauma	N/A	Partial defect, alive	AR: N/A, CR: No	N/A
Bettoni et al. [23]	September of 2012	Amiens, France	N/A	Vascular tumor	Eating	Partial defect, alive	AR: Yes, CR: No	N/A
Chandraker et al. [25]	February of 2013	Boston, Massachusetts	44 y	Chemical burn	Speech	Full face defect, alive	AR: Yes, CR: Yes (re-transplanted)	At 12 months: Mental health QoL stable, At 18 months: Mental health quality worsened, at 9-24 months: Physical health quality improved, Normal-to-high self-esteem
Krakowczyk et al. [28]	December of 2013	Gliwice, Poland	28 y	Neurofibromatosis	Speech, eating	Full face defect, alive	AR: Yes, CR: No	Saw face first on day 9, gradually accepted it, no adaptive or psychiatric problems
Knackstedt et al. [31]	May of 2017	Cleveland, Ohio	28 y	Ballistic trauma	Ocular and periocular competence, speech, swallowing, nasal breathing, tongue movement, oro-myofunctional	Full face defect, alive	AR: Yes, CR: No	N/A
Santanelli et al. [32]	September of 2018	Rome, Italy	21 y	Neurofibromatosis	Left facial nerve palsy, ocular and periocular competence, eating, oro-myofunctional competence	Partial defect, alive	Hyperacute rejection, she is on the transplantation waitlist	N/A
Kauke et al. [30]	July of 2020	Boston, Massachusetts	52 y	CR of previous face transplantation	Speech	Full face defect, alive	AR: Yes	N/A

Ninety-six patients received 150 hand transplants (44 unilateral and 53 bilateral). Of the 96 reported hand and upper extremity transplants, 20 recipients were female cases, 11 of whom underwent bilateral limb transplants. Sixteen (10.8%) of the upper extremity transplants were amputated after follow-up with the most common reason being the discontinuation of immunosuppressive drugs [33]. To date, no graft loss has been observed due to AR while on triple-drug immunosuppressive therapy [34]. However, when face transplantation is combined with limb transplantation, the mortality rate increases [35,36]. Except for the Chinese patient who discontinued immunosuppression and follow-up in a face transplantation case, no other patients have been lost. Table 2 summarizes hand transplant cases in women performed until 2024 [10,37-58]. Recently, the Indian experience published by Kadam mentioned an explanation of hand allograft in two patients [58]; among them was a female who received a double-hand transplant (Janarthanan R, Personal Communication, 2025). 

**Table 2:** Female hand transplantation until 2024. AR: acute rejection, CR: chronic rejection.

Surgical team	Year	Location	Age	Indication	Unilateral/ bilateral	Level of transplant	Updated information	Rejection	Psychosocial evaluation/patient testimony
Pei et al. [37]	2003	China	37 y	Mechanical accident	Unilateral	Proximal forearm	The graft was lost in October 2024, due to non-compliance with an immunosuppressive regimen and subsequent rejection and necrosis of allograft	AR: Yes, CR: N/A	The pre-transplant psychosocial evaluation was done
Landin et al. [39]	2006	Spain	47 y	Explosion	Bilateral	N/A	At 20 months, sensory recovery was good but not complete. Muscle recovery was strong in the left hand but poor in the right hand and needed tendon transfers	AR: Yes, (2 episodes), CR: N/A	Psychiatric evaluation did not reveal any problems, and the patient showed a positive and realistic approach to transplantation
Petruzzo et al. [40]	2007	France	27y	Electrocution	Bilateral	Mid-forearm	N/A	AR: Yes (6 episodes)	N/A
Chełmoński et al. [41]	2010	Poland	N/A	Circular saw	Unilateral	Mid-forearm	At the 36-month follow-up, the patient was able to bend the elbow 50° against gravity. Sensation for pain, temperature, and protection extended to the fingertips, with only mild intolerance to cold	N/A	Greater self-confidence, higher self-esteem. Socially active in non-profit organizations
Schneeberger et al. [42]	2010	USA	25 y	Norovirus sepsis	Unilateral	Distal forearm	N/A	AR: Yes (on day 18, responded to steroid), CR: N/A	N/A
Schneeberger et al. [42]	2010	USA	33 y	Meningococcal sepsis	Bilateral	Distal forearm	N/A	AR: Yes (on day 51, responded to topical tacrolimus/clobetasol), CR: N/A	N/A
Jablecki et al. [44]	2010	Poland	55 y	Motor vehicle accident	Unilateral	Mid-arm	At 19 months post-transplant, the patient can flex her elbow to 75° against gravity. She has a full finger range of motion (180°), protective sensation in the fingertips, and functioning radial nerve sensation. The patient can distinguish thermal stimuli, has mild cold intolerance, and normal perspiration	At 19 months follow up, no AR or CR has been observed	She is very satisfied with the outcome, particularly appreciating the close aesthetic match between both hands
Tuder et al. [45]	2010	USA	58 y	Package bomb blast	Unilateral	Mid/distal one-third forearm	After 9 months, there is a progressive Tinel's sign at the interphalangeal level of the thumb and the proximal interphalangeal level of the other fingers. The separation of hot and cold in the palm of the hand has returned	N/A	The patient uses the transplanted limb for several daily activities such as cooking, writing, and opening doors. She also resumed horseback riding with minimal difficulty
Surgical team led by Dr. Azari [46]	2011	USA	26 y	N/A	Unilateral	Hand	N/A	N/A	N/A
Surgical team led by Dr. Levin [47]	2011	USA	N/A	N/A	Bilateral	N/A	N/A	N/A	N/A
Surgical team led by Dr. Cendales [48]	2011	USA	21 y	N/A	Unilateral	N/A	N/A	N/A	Patient testimony: "Right now I am filled with emotions, and I don't think that there is really anything that can describe how I fell, other than just happiness, hopefulness, and of course thankfulness."
Del Bene et al. [10]	2011	Italy	52 y	Sepsis	Bilateral	N/A	N/A	N/A	N/A
Surgical team led by Dr. Pomahac [49]	2011	USA	57 y	Chimpanzee attack	Bilateral	R: Mid-carpus; L: Mid-forearm	The hand allograft was explanted due to complications	N/A	N/A
Cendales et al. [50]	2012	USA	21 y	Kawasaki disease	Unilateral	Distal forearm	At 1-year follow-up, the patient reported good compliance with the transplant. Tinel's sign was present at the fingertips, and she was able to perform daily activities using both hands. Clinical examination revealed no noticeable changes in allograft coloration or dysfunction in terms of range of motion and sensation	AR: No, CR: N/A	N/A
Adler et al. [51]	2014	USA	33 y	Meningococcemia, gangrene	Bilateral	Mid-forearm	The immunotherapy regimen consisted of a bone marrow cell infusion from the donor and maintenance tacrolimus monotherapy	AR: No, CR: N/A	N/A
Ben-Amotz et al. [52]	2016	USA	28 y	Streptococcal sepsis	Bilateral	Arm below the elbow at the proximal forearm	At 8-month follow-up, the motor examination showed strength scores exceeding expectations. Sensory function is gradually improved with restored perception of hot and cold stimuli	N/A	The patient remained highly active despite physical limitations and had a clear understanding of the surgery's risks and benefits. Preoperative psychological evaluations were done
Surgical team led by Dr. Ozyurekoglu [53]	2016	USA	69 y	Infection	Bilateral	Hand	N/A	N/A	Patient testimony: ‘’It is truly a miracle that I received these new hands’’
Surgical team led by Dr. McCabe [54]	2016	Canada	50 y	Car accident	Unilateral	Below elbow	N/A	N/A	After the surgery she was scared but then after the recovery she said “It's really changed my life. I'm whole again”
Surgical team led by Dr. Iyer [55]	2017	India	19 y	Bus accident	Bilateral	above elbow	She is currently on rehabilitation. Authors expect that she will regain 85 percent of hand function in the next one and a half years	N/A	She was satisfied. She expressed herself “My whole world collapsed, and I couldn’t believe what had happened… and my disability began to look temporary...”
Surgical team led by Dr. Cendales [56]	2018	USA	N/A	Streptococcal infection	Bilateral	hand	N/A	N/A	She was very satisfied. She expressed herself ‘’It’s just beyond my expectations”
Kadam D [58]	2024	India	45 y	Workplace accident	Bilateral	Arm	Both hand allografts were explanted	N/A	N/A

A separate study involving the recruitment of 72 candidates for face transplantation found that most participants were men with burn injuries, while 31 were women. Although referral rates were nearly evenly split between physicians and self-referrals, physician-referred patient contacts were mostly male, and self-referred patients were female. Individuals with burns, gunshot wounds, and congenital defects were likely to be referred by their physicians and then self-referred [59].

According to one survey study with consulting anesthesiologists, from the perioperative evaluation perspective, no sex or gender-specific distinctions or requirements were found among the 14 face transplant cases [60].

Sex and gender-specific differences in male and female transplantation

Anatomic and Physiologic Differences in Male and Female Recipients

Specific surgical considerations for hand and face transplants, such as unique anatomy (anthropometric and cephalometric differences in dimensions), require careful donor-recipient matching and customized surgical techniques to achieve optimal functional and aesthetic outcomes. 

An understanding of the anatomical differences in VCA is critical to the tailoring of surgical techniques and post-operative care, all of which are key to achieving a natural and harmonious integration post-transplant. Male individuals typically have larger and denser bones and more muscle mass than female individuals which affects the size and type of graft needed, especially for limb transplants [61]. Matching cephalometric parameters could be critical in facial transplants to ensure precise anatomical alignment and symmetry between the donor and recipient, which is vital for functional and aesthetic outcomes. In this regard, sex matching is essential as male and female individuals have unique craniofacial proportions, soft tissue thickness, and skeletal structure.

Vessels in male individuals tend to be larger [62] which makes anastomosing technically simpler, while differences in blood flow dynamics can affect healing and the risk of complications such as thrombosis. Male individuals often have thicker skin with more collagen [63] while female individuals have more subcutaneous fat [64], which has an impact on skin healing and graft adaptation. We can envision VCA allotransplantation across sexes (male → female or female → male) in the event of non-bony allografts, especially when size and color matching are accomplished without concerns for hair-bearing skin.

Despite the expected vascular advantages in male individuals, postoperative complications like congestion, edema, and ischemic events were not more common in women. This suggests that male anatomy does not significantly impact these outcomes as initially anticipated [1]. There is no mention of technical issues, such as vascular complications, or any vascular differences between the sexes [65].

Functional outcomes and rehabilitation needs also differ, with males and females requiring tailored rehabilitation based on anatomical and physiological differences. Recognizing these sex and gender differences is essential for successful VCA outcomes, allowing for precise surgical planning, tailored post-operative care, and improved outcomes for both males and females.

Hormonal Differences in Male and Female Recipients

The hormonal influences on VCAs, which involve the transplantation of multiple tissue types such as skin, muscle, bone, and blood vessels, are critical to elucidating outcomes including rejection. Given the complexity of VCAs compared to single organ transplants, understanding these hormonal influences on integration, survival, and function is essential to improve outcomes and increase the success of VCA procedures.

In female patients, hormonal changes during the menstrual cycle and pregnancy can impact immune response and graft stability, necessitating close monitoring and adjustments to immunosuppressive therapy. In contrast, male patients generally experience more stable hormone levels, resulting in more consistent immune responses and graft integration.

Androgens can prolong inflammation and inhibit wound healing, while estrogens reduce inflammation via anti-inflammatory cytokines and promote wound healing by increasing collagen synthesis and angiogenesis. In one animal study, testosterone was found to negatively impact wound healing, potentially disrupting re-epithelialization and altering the inflammatory balance [66]. Despite these known effects, no animal studies have yet evaluated the impact of hormones on wound healing in sex-mismatched transplants.

In female patients, hormonal changes during the menstrual cycle and pregnancy can impact immune response and graft stability, necessitating close monitoring and adjustments to immunosuppressive therapy. Pregnancy can also increase the panel reactive antibody (PRA) levels in female patients awaiting VCA due to sensitization caused by exposure to fetal antigens, which can trigger an immune response and lead to the development of anti-donor HLA antibodies after transplantation. In contrast, male patients generally experience more stable hormone levels, resulting in more consistent immune responses and graft integration.

Female patients who have undergone transplantation have no specific contraindications for hormonal therapy. However, due to the increased likelihood of early menopause and the higher risks of cardiovascular disease and bone density loss associated with long-term immunosuppression, hormonal therapy is generally recommended [67]. This is especially true for female patients on long-term steroid immunosuppression as they can disrupt the hypothalamic-pituitary-gonadal (HPG) axis, leading to irregular menstrual cycles or cause secondary amenorrhea due to suppression of gonadotropin-releasing hormone (GnRH) secretion. Finally, drugs like mycophenolate mofetil (Cellcept), commonly used in VCA, could be teratogenic and lead to miscarriages in female recipients who may become pregnant after transplantation.

Immunological Impact of Sex and Gender

Thanks to advanced microsurgical techniques and low complication rates in surgery [68], progress in VCA is closely linked to advances in immunology. Advances in immunology have led to the development of immunomodulatory drugs by enhancing our understanding of rejection reactions, much like how the discovery of ABO blood groups [69] laid the foundation for tissue transplantation. In VCA, as in SOT, AR is characterized by lymphocyte infiltration observed in biopsy [70] within the first two weeks after transplantation. This can be effectively prevented with pulsed steroids [8,71]. According to the International Registry for Hand and Composite Tissue Transplantation, 80% of recipients experience at least one AR episode within the first 12 months, making close monitoring and effective immunosuppressive management crucial to preventing graft failure and ensuring long-term transplant viability [44] (Table 2). In their review, Van Dieren et al. report that AR is more common among male patients, burn patients, those who receive upper extremity transplants, and individuals over 30 years of age-old [72]. Furthermore, since some AR cases are associated with cytomegalovirus (CMV) infection, CMV prophylaxis should be carefully considered [73].

Immunological challenges in transplantation arise even before the procedure begins. Many recipients have high levels of panel reactive antibodies (PRA) in the blood due to repeated blood transfusions and transplant history prior to transplantation, making an acceptable gender-specific immunological match difficult. Hence, cross-sex (CS)-VCA could lead to a larger donor pool, shorter waiting lists, and a better chance of finding a compatible match, especially for patients with high sensitivity [74]. To date, only five cases of CS-VCA (all involving extremities) have been reported, with limited follow-up (≤2 years), but long-term outcomes including rejection have not been reported [43,75-79]. Despite significant advances since the last attempt at CS-VCA in 2011, minimizing the risk of immunological compromise remains essential for success [80]. This requires careful donor selection, thorough surgical rehearsal and planning to reduce intraoperative ischemia time, and careful rejection monitoring coupled with timely and appropriate treatment [80].

CR, reported in 11% of VCA recipients, including face and upper limb transplants, is often associated with patient non-compliance [15]. Traditionally, B cells play a role in solid organ transplantation by producing donor-specific antibodies, which contribute to CR through vasculopathy and ultimately lead to transplant failure [81,82]. In contrast, possibly due to its unique immunological environment that promotes graft adaptation and tolerance, VCA, which includes skin, muscle, and bone, induces less antibody-mediated rejection compared to solid organ transplants [83]. According to Huelsboemer et al., antibody-mediated injury appears to be less critical in face transplantation [84,85]. Since most donor-specific antibodies are produced through T cell-dependent B cell responses, VCA immunosuppression primarily targets T cells [83]. There is a female case where a retransplanted individual developed donor-specific antibodies after the first rejection [85]. 

The buccal mucosa may be complementary to routine skin biopsies in facial VCA, but its value is limited by the absence of a standardized grading system for AR, unlike the Banff Grading used for skin. Mucosal biopsies reveal CD4+ T cells, regulatory T cells (Tregs), macrophages, and occasionally eosinophils in histopathological analysis. However, the mucosa's dynamic environment-characterized by continuous exposure to pathogens, mechanical stress, and baseline inflammation-can influence these findings and may lead to variability in interpretation [86]. Furthermore, mucosal biopsies carry risks such as bleeding, pain, and infection due to the oral cavity's dense microbial flora, which limits their practicality [30,87]. Notably, some studies exclude mucosal biopsy results, highlighting the challenges associated with their interpretation and consistency [85]. Despite these limitations, the pronounced inflammatory changes in mucosal biopsies compared to skin biopsies may provide a valuable indicator for predicting CR.

Despite the role of estrogen as an immunomodulatory hormone, females generally have higher CD4+ T cell counts, CD4/CD8 ratios, and greater numbers of activated CD4+ and CD8+ T cells, as well as stronger antibody responses and higher B cell counts compared to males [88]. They also produce higher levels of both TH1-type and TH2-type cytokines, though IL-17 production varies depending on conditions. In contrast, males exhibit higher frequencies of CD8+ T cells and Treg cells [89]. Macrophages also play a key role in immune system responses. In a macrophage-targeted nanotheranostics study conducted by Liu et al., CFA-induced inflammation was more effectively reduced in male versus female mice [90].

From our understanding of physiology and autoimmune diseases, women tend to exhibit more pronounced immunological responses. The stronger immune response in females may provide better protection against infections, vaccines, and cancer, making immunotherapies more effective in female individuals [91,92]. In the case of Graft versus Host Disease in VCA, regulatory T cells play a key role in its pathophysiology [93], like autoimmune diseases like Crohn's disease and ulcerative colitis [94]. In a study of 904 patients with complete medical histories, gender differences in treatment strategies for Crohn's disease and ulcerative colitis were analyzed, revealing that female patients were prescribed fewer immunosuppressive medications compared to their male counterparts [95].

In the world experience to date, there is limited data to confirm that rejection is more severe in women undergoing VCA. The first reported case of facial re-transplantation in a female patient stands out due to its exceptionally complex immunological challenges. This unique case involved high sensitization (PRA 98%) and a positive crossmatch during the initial transplant, ultimately leading to CR and graft loss, despite antibody-mediated rejection being relatively rare in VCA compared to SOT [30,85]

The pharmacokinetics of immunosuppressive drugs can vary between male and female organ transplant recipients due to differences in body composition, hormone levels, and enzyme activity. Female individuals generally have higher body fat, affecting the distribution of lipophilic drugs such as tacrolimus (Prograf) or rapamycin (Sirolimus), while male individuals have higher lean body mass, influencing dosing of hydrophilic drugs such as mycophenolic acid (Cellcept).

Hormonal differences, particularly androgens in males and estrogen in females can modulate liver enzyme activity (e.g., cytochrome P450), altering the metabolism and clearance of certain drugs, such as tacrolimus and mycophenolate mofetil, potentially leading to variations in drug efficacy and toxicity between genders [96]. In male individuals, testosterone enhances cytochrome P450 (CYP3A4) activity, which metabolizes many immunosuppressants like tacrolimus and rapamycin, thereby increasing drug metabolism and potentially lowering drug levels, which may reduce efficacy. Testosterone also influences drug transporters like P-glycoprotein and may reduce drug distribution and clearance. On the other hand, estrogens often inhibit CYP3A4, increasing drug concentrations and toxicity risks, while progestins have variable effects on drug metabolism. Both hormones can alter transporter activity, impacting drug distribution and excretion. These interactions necessitate careful monitoring, particularly in scenarios like hormonal therapy or pregnancy, to optimize drug efficacy and minimize adverse effects

Immunosuppression in VCA recipients leads to various complications, including opportunistic infections (58% overall, 68% in face transplant patients), metabolic disorders like diabetes and hyperlipidemia, and rare occurrences of malignancies, avascular necrosis, and serum sickness [15,71]. Recent research suggests that mesenchymal stem cells could be a promising alternative, reducing rejection and improving outcomes. For example, Tan et al. found lower rejection rates and better outcomes in kidney transplants using mesenchymal stem cells compared to conventional methods [97]. Chimerism, the adaptation of the immune system to new tissue, is crucial for tolerance [98,99], with even 1% [34]. Outcomes of the Pittsburgh Protocol, comprising of a donor bone marrow infusion and tacrolimus monotherapy in a series of five hand transplant recipients performed at the University of Pittsburgh have shown mixed results with only some patients being successfully maintained on monotherapy [34, 100].

Impact of Sex and Gender on Psychosocial Outcomes

Biological sex plays a critical role in surgical planning, post-operative healing, and immunologic outcomes, while gender significantly influences self-identity and body image adaptation in patients undergoing hand and face transplants. Gender identity shapes how patients perceive, internalize, and cope with their transplants, directly affecting psychosocial outcomes. Together, sex and gender have a profound impact on post-transplant adjustment and societal perception. Unlike SOT, where sex-mismatched transplants are common and rarely affect psychosocial outcomes, the outward visibility of sex-based features in hand and face transplants introduces unique challenges. Mismatched transplants involving visible characteristics, such as masculine or feminine facial traits or hands, can lead to stigma, particularly due to societal expectations. For women, these expectations may heighten psychosocial stress, amplifying the impact of disfigurement while also enhancing the relief experienced post-transplant [85,101].

Gender norms further impose pressures, including beauty standards for women and functionality expectations for men. Recovery is also influenced by societal norms, with female patients often facing distinct challenges such as heightened emphasis on aesthetic outcomes and social reintegration. Quality of life improves with strong social and psychiatric support, while aesthetic satisfaction is shaped by factors like injury severity, gender, and personal expectations. Women may experience greater social pressure and hold higher aesthetic expectations [15].

Facial disfigurement causes as many sociopsychological problems as a physical disability [16]. Social isolation often leads to various psychiatric issues, including addiction, anxiety disorders, and depression [102]. Face transplantation has been a pivotal intervention for improving these outcomes. For instance, in one case, a female patient reported significantly reduced depression and fewer instances of verbal abuse following her face transplant [103]. Hadjiandreou et al. demonstrated that a gradual psychosocial recovery and integration into the family and community environment were reported as significant benefits [14].

As the prevalence of VCA has grown, so has interest in the psychological effects of these procedures. While SOT often leads to improved physical health, it also introduces psychological challenges, including anxiety about organ rejection, long-term immunosuppression, and the demands of ongoing graft care [104,105]. These include anxiety related to potential organ rejection, the long-term effects of immunosuppression, and the personal responsibilities involved in graft care. In the case of face transplantation, issues of communication and identity perception often arise [71]. The acceptance of a new face may induce a grief-like state, as individuals grapple with the loss of their previous identity and the challenge of adjusting to a new one [106].

Older patients, particularly women, have demonstrated better management strategies, relying more on emotional and instrumental support. Pre-existing mental health conditions, such as depression or post-traumatic stress disorder, which are common in recipients of both sexes, can influence psychological recovery, with women sometimes reporting greater emotional distress [106].

Public attitudes toward face transplantation vary, but a large survey conducted in Great Britain revealed that most people were unconcerned about the procedure [107]. However, women expressed significantly higher levels of concern compared to men. In the survey of 2,122 individuals, including 1,024 women, respondents with facial differences were more likely to view the psychological impact of disfigurement as a valid reason for considering face transplantation. Public awareness of the psychological benefits of face transplants is increasing but concerns about long-term outcomes and ethical considerations persist [107].

A multicenter study conducted between July 2020 and March 2022 interviewed 50 candidates with upper limb loss to assess perceptions of selection criteria for upper limb VCA. The study found that candidates, 11 of whom were female individuals, identified factors such as young age, good physical health, mental stability, motivation, and strong social support as essential for successful transplantation. These criteria align with current transplant center practices, which prioritize medical and psychological readiness. However, the study also highlighted certain limitations, including the predominantly male and White individuals, as well as the lack of participants’ understanding of the risks associated with upper limb VCA such as long-term immunosuppression. Many participants believed that individuals with bilateral limb loss would benefit more from the procedure compared to those with unilateral loss [108].

This creates a conflict with the medical principle of "first, do no harm." Until immunosuppressive therapies can be further optimized to reduce adverse effects, this ethical dilemma will persist. Patient selection for face and upper extremity transplantation is currently guided by the anticipated postoperative benefits, with a particular focus on the patient’s psychological readiness. Psychological development is often the most critical determinant of long-term success in these procedures, as well as the ability to comply with lifelong immunosuppression [65].

Both sexes initially see improvements in quality of life, but these benefits often fade over time due to medical complications and the mental strain of lifelong immunosuppression [101]. Today, patient selection for face and upper extremity transplantation is primarily guided by the anticipated level of benefit in the postoperative period, with the patient's psychological development being the most significant determinant of that benefit.

Sex and Gender-Based Differences in Functional Outcomes

Unlike popular belief, surgical decisions for VCA candidates are not based entirely on aesthetic and psychosocial outcomes. In facial transplantation, critical functional outcomes include the ability to breathe, eat, speak, express facial movements, and experience facial sensation [109,110]. Similarly, in upper extremity transplantation, functional success is evaluated based on the patient’s ability to perform daily tasks [8].

For hand transplantation, the International Hand Transplant Score System (IHTSS) scoring system emphasizes key markers such as the appearance of the grafted hand, skin color, hair and nail growth, and vascularization [111]. Motor recovery-the restoration of mobility-is another critical factor in assessing outcomes. Both motor and sensory functions typically improve within the first five years after transplantation, whether in bilateral or unilateral limb transplants. A follow-up study involving 30 patients (including two women) over a period of one year (1998-2007) revealed annual improvements in mobility, although no significant differences were observed between male and female patients [8].

According to Wollesen et al., incorporating gender-specific insights into exoskeleton design can help reduce discomfort and increase usability, particularly for female users in occupational and therapeutic settings [112]. Gender-inclusive approaches are essential to optimize the effectiveness and user experience of assistive devices and ensure broader applicability to diverse populations [112]. 

Tacrolimus, the keystone drug used in VCA, has a complementary effect on improving nerve regeneration (via its activity as a no-immunosuppressant FKBP-12 ligand), as shown in studies involving isolated nerve allografts [113]. In VCA, unlike in SOT, regeneration of nerves is key to functional outcomes. In other words, a viable VCA could still be non-functional due to a lack of nerve regeneration. Tacrolimus increases the count of myelinated fibers, accelerates axonal growth cone advancement, and enhances reinnervation. However, the recovery of extrinsic muscle function largely depends on whether the native forearm muscles have been preserved or replaced during transplantation [114].

In addition to tacrolimus, growth hormone (GH) plays an important role in nerve regeneration. GH accelerates axonal regeneration, promotes myelination, reduces muscle atrophy, and enhances muscle reinnervation [115]. Resting GH secretion is significantly higher in young female individuals compared to males of the same age, potentially offering females an advantage in nerve repair processes [116]. Both GH and tacrolimus are metabolized via the same P450 pathway, and since tacrolimus is known to positively impact nerve healing, it can be challenging to distinguish whether the improvement in nerve function in VCA patients is due to GH or tacrolimus [117].

When evaluating functional outcomes and peripheral nerve injuries, research consistently shows that sex and gender do not play a significant role in the recovery process. Studies across multiple cohorts confirm that factors such as health and surgical techniques have a greater impact on recovery than sex or gender [14,34,108-110,118]

In summary, the timeline and critical phases involved in the recovery and risks associated with hand and face transplants vary between female and male recipients. The key differences lie in re-perfusion injury, wound healing, recovery of motor and sensory function, and aging and atrophy of the graft, due to the short and long-term effects of AR or CR.

## Conclusions

While the role of sex and gender in VCA is gaining attention, integrating it into the clinical practice of VCA remains a challenge. The lack of large, controlled studies examining sex and gender as key variables limits the generalizability of findings, particularly for procedures such as hand and face transplantation, where case numbers are low worldwide. Biological factors, including hormonal variations and immune responses, are known to influence graft adaptation, function, and rejection, but their mechanisms and interactions with immunosuppressive therapies remain poorly understood in CS-VCA.

In addition, gender-specific psychosocial factors, such as emotional readiness and social support, are difficult to measure and address, creating barriers to tailored psychological care. Future research is needed to explore the multifaceted impact of sex and gender on donor selection, surgical planning, and post-transplant care. Improving this understanding will help to improve surgical, immunological, psychosocial, and quality of life outcomes for VCA recipients.

## References

[1] Devauchelle B, Badet L, Lengelé B (2006). First human face allograft: early report. Lancet.

[2] Dubernard JM, Owen E, Herzberg G (1999). Human hand allograft: report on first 6 months. Lancet.

[3] Lewis HC, Cendales LC (2021). Vascularized composite allotransplantation in the United States: a retrospective analysis of the Organ Procurement and Transplantation Network data after 5 years of the Final Rule. Am J Transplant.

[4] Siemionow MZ, Zor F, Gordon CR (2010). Face, upper extremity, and concomitant transplantation: potential concerns and challenges ahead. Plast Reconstr Surg.

[5] Jochum F, Hamy AS, Gougis P (2024). Sex-related differences in oncological surgery and postoperative outcomes: comprehensive, nationwide study in France. Br J Surg.

[6] Unger JM, Vaidya R, Albain KS (2022). Sex differences in risk of severe adverse events in patients receiving immunotherapy, targeted therapy, or chemotherapy in cancer clinical trials. J Clin Oncol.

[7] Alberti FB, Hoyle V (2021). Face transplants: an international history. J Hist Med Allied Sci.

[8] Petruzzo P, Lanzetta M, Dubernard JM (2008). The international registry on hand and composite tissue transplantation. Transplantation.

[9] Thuong M, Petruzzo P, Landin L (2019). Vascularized composite allotransplantation - a Council of Europe position paper. Transpl Int.

[10] Del Bene M, Di Caprio AP, Melzi ML, Pioltelli PE, Bonomi S (2013). Autologous mesenchymal stem cells as a new strategy in immunosuppressant therapy in double hand allotransplantation. Plast Reconstr Surg.

[11] Mendenhall SD, Ginnetti MT, Sawyer JD, Verhulst SJ, West BL, Levin LS, Neumeister MW (2018). Prevalence and distribution of potential vascularized composite allotransplant donors, implications for optimizing the donor-recipient match. Plast Reconstr Surg Glob Open.

[12] Wainright JL, Wholley CL, Cherikh WS, Musick JM, Klassen DK (2018). OPTN vascularized composite allograft waiting list: current status and trends in the United States. Transplantation.

[13] Rezwan SK, Puthumana JS, Brandacher G, Cooney CM (2024). Crowdsourcing attitudes and beliefs about upper extremity donation in the United States. Cureus.

[14] Hadjiandreou M, Pafitanis G, Butler PM (2024). Outcomes in facial transplantation - a systematic review. Br J Oral Maxillofac Surg.

[15] Milek D, Reed LT, Echternacht SR (2023). A systematic review of the reported complications related to facial and upper extremity vascularized composite allotransplantation. J Surg Res.

[16] Petruzzo P, Testelin S, Kanitakis J (2012). First human face transplantation: 5 years outcomes. Transplantation.

[17] Guntinas-Lichius O (2008). Outcomes 18 months after the first human partial face transplantation. N Engl J Med.

[18] Morelon E, Petruzzo P, Kanitakis J (2017). Face transplantation: partial graft loss of the first case 10 years later. Am J Transplant.

[19] Siemionow MZ, Papay F, Djohan R (2010). First U.S. near-total human face transplantation: a paradigm shift for massive complex injuries. Plast Reconstr Surg.

[20] Bergfeld W, Klimczak A, Stratton JS, Siemionow MZ (2013). A four-year pathology review of the near total face transplant. Am J Transplant.

[21] Pomahac B, Pribaz J, Eriksson E (2012). Three patients with full facial transplantation. N Engl J Med.

[22] Ozmen S, Findikcioglu K, Sibar S (2023). First composite woman-to-woman facial transplantation in Turkey: challenges and lessons to be learned. Ann Plast Surg.

[23] Bettoni J, Balédent O, Petruzzo P (2020). Role of flow magnetic resonance imaging in the monitoring of facial allotransplantations: preliminary results on graft vasculopathy. Int J Oral Maxillofac Surg.

[24] Chandraker A, Arscott R, Murphy G (2016). Face transplantation in a highly sensitized recipient. Mil Med.

[25] Chandraker A, Arscott R, Murphy GF (2014). The management of antibody-mediated rejection in the first presensitized recipient of a full-face allotransplant. Am J Transplant.

[26] Diaz-Siso JR, Sosin M, Plana NM, Rodriguez ED (2016). Face transplantation: complications, implications, and an update for the oncologic surgeon. J Surg Oncol.

[27] Win TS, Murakami N, Borges TJ (2017). Longitudinal immunological characterization of the first presensitized recipient of a face transplant. JCI Insight.

[28] Krakowczyk Ł, Maciejewski A, Szymczyk C, Oleś K, Półtorak S (2017). Face transplant in an advanced neurofibromatosis type 1 patient. Ann Transplant.

[29] Nizzi MC, Tasigiorgos S, Turk M, Moroni C, Bueno E, Pomahac B (2017). Psychological outcomes in face transplant recipients: a literature review. Curr Surg Rep.

[30] Kauke M, Panayi AC, Safi AF (2021). Full facial retransplantation in a female patient-technical, immunologic, and clinical considerations. Am J Transplant.

[31] Knackstedt R, Siemionow M, Djohan R (2022). Youngest composite full-face transplant: a model for vascularized composite allograft in younger populations. Ann Plast Surg.

[32] Santanelli di Pompeo F, Longo B, Giovanoli P (2021). Facial transplantation: nonimmune-related hyperacute graft failure-the role of perfusion injury: a case report. Ann Plast Surg.

[33] Wells MW, Rampazzo A, Papay F, Gharb BB (2022). Two decades of hand transplantation: a systematic review of outcomes. Ann Plast Surg.

[34] Brandacher G, Gorantla VS, Lee WP (2010). Hand allotransplantation. Semin Plast Surg.

[35] Schneeberger S, Gorantla VS, van Riet RP (2008). Atypical acute rejection after hand transplantation. Am J Transplant.

[36] Shores JT, Lee WP, Brandacher G (2013). Discussion: lessons learned from simultaneous face and bilateral hand allotransplantation. Plast Reconstr Surg.

[37] Pei G, Xiang D, Gu L (2012). A report of 15 hand allotransplantations in 12 patients and their outcomes in China. Transplantation.

[38] Cavadas PC, Landin L, Ibañez J (2009). Bilateral hand transplantation: result at 20 months. J Hand Surg Eur Vol.

[39] Landin L, Cavadas PC, Nthumba P (2010). Morphological and functional evaluation of visual disturbances in a bilateral hand allograft recipient. J Plast Reconstr Aesthet Surg.

[40] Petruzzo P, Gazarian A, Kanitakis J (2015). Outcomes after bilateral hand allotransplantation: a risk/benefit ratio analysis. Ann Surg.

[41] Chełmoński A, Kowal K, Jabłecki J (2015). The physical and psychosocial benefits of upper-limb transplantation: a case series of 5 Polish patients. Ann Transplant.

[42] Schneeberger S, Gorantla VS, Brandacher G (2013). Upper-extremity transplantation using a cell-based protocol to minimize immunosuppression. Ann Surg.

[43] Jabłecki J (2011). World experience after more than a decade of clinical hand transplantation: update on the Polish program. Hand Clin.

[44] Jablecki J, Kaczmarzyk L, Domanasiewicz A (2012). Result of arm-level upper-limb transplantation in two recipients at 19- and 30-month follow-up. Ann Transplant.

[45] Tuder D, Pederson WC, Abrahamian GA, Ingari JV, Bagg MC, Person DW, Martyak GG (2011). San Antonio military and civilian hand transplantation program: a case report. Transplant Proc.

[46] (2025). UCLA performs first hand transplant in the western United States. https://www.uclahealth.org/news/release/ucla-performs-first-hand-transplant-in-the-western-united-states.

[47] (2025). UPenn’s double hand transplant. https://www.phillymag.com/news/2011/11/01/upenns-double-hand-transplant/.

[48] (2025). Rare hand transplant surgery successfully performed at Emory University Hospital. https://medicalxpress.com/news/2011-03-rare-transplant-surgery-successfully-emory.html.

[49] (2025). Victim of chimp attack gets a full face transplant. https://www.cbs8.com/article/news/victim-of-chimp-attack-gets-a-full-face-transplant/509-30eb6584-a411-4f3a-a512-f37cd930f5f9.

[50] Cendales L, Bray R, Gebel H (2015). Tacrolimus to belatacept conversion following hand transplantation: a case report. Am J Transplant.

[51] Adler BL, Albayda J, Shores JT, Lee WP, Brandacher G, Bingham CO 3rd (2017). Erosive rheumatoid arthritis after bilateral hand transplantation. Ann Intern Med.

[52] Ben-Amotz O, Kruger EA, McAndrew C (2018). Logistics in coordinating the first adult transatlantic bilateral hand transplant: lessons learned. Plast Reconstr Surg.

[53] (2025). Double hand transplant patient released from Jewish Hospital. https://www.businesswire.com/news/home/20161123005556/en/Double-Hand-Transplant-Patient-Released-from-Jewish-Hospital..

[54] (2025). Canada's first hand transplant patient says surgery has made her 'whole' again. https://www.cbc.ca/news/science/first-hand-forearm-transplant-1.3649102.

[55] (2025). India's first such transplant gives 19-year-old her hands back. https://yourstory.com/2017/10/first-hand-transplant-india.

[56] (2025). Duke Health team performs first bilateral hand transplant on Thanksgiving Day. https://surgery.duke.edu/news/duke-health-team-performs-first-bilateral-hand-transplant-thanksgiving-day.

[57] (2025). The voice of VCA patients. Patient stories. https://worldofvca.com/patient-stories/.

[58] Kadam D (2024). Indian plastic surgery teams lead with the world's highest number of hand transplants. Indian J Plast Surg.

[59] Kiwanuka H, Aycart MA, Bueno EM, Alhefzi M, Krezdorn N, Pomahac B (2016). Patient recruitment and referral patterns in face transplantation: a single center's experience. Plast Reconstr Surg.

[60] Edrich T, Cywinski JB, Colomina MJ (2012). Perioperative management of face transplantation: a survey. Anesth Analg.

[61] Lang TF (2011). The bone-muscle relationship in men and women. J Osteoporos.

[62] Beale AL, Meyer P, Marwick TH, Lam CS, Kaye DM (2018). Sex differences in cardiovascular pathophysiology: why women are overrepresented in heart failure with preserved ejection fraction. Circulation.

[63] Seidenari S, Pagnoni A, Di Nardo A, Giannetti A (1994). Echographic evaluation with image analysis of normal skin: variations according to age and sex. Skin Pharmacol.

[64] Li L, Zhou Z, Fang J (2024). The characterization of metabolic changes in adipose tissues and muscles due to different exercise intensities by Dixon in healthy young men. Eur J Radiol.

[65] Siemionow M, Ozturk C (2012). Face transplantation: outcomes, concerns, controversies, and future directions. J Craniofac Surg.

[66] Reiche E, Tan Y, Louis MR (2022). A novel mouse model for investigating the effects of gender-affirming hormone therapy on surgical healing. Plast Reconstr Surg Glob Open.

[67] Cyganek A, Pietrzak B, Wielgoś M, Grzechocińska B (2016). Menopause in women with chronic immunosuppressive treatment - how to help those patients. Prz Menopauzalny.

[68] Petruzzo P, Sardu C, Lanzetta M, Dubernard JM (2017). Report (2017) of the International Registry on Hand and Composite Tissue Allotransplantation (IRHCTT). Curr Transpl Rep.

[69] Landsteiner K, Chase MW (1942). Experiments on transfer of cutaneous sensitivity to simple compounds. Proc Soc Exp Biol Med.

[70] Alolabi N, Augustine H, Thoma A (2017). Hand transplantation: current challenges and future prospects. Transplant Res Risk Manag.

[71] Morris P, Bradley A, Doyal L, Earley M, Hagen P, Milling M, Rumsey N (2007). Face transplantation: a review of the technical, immunological, psychological and clinical issues with recommendations for good practice. Transplantation.

[72] Van Dieren L, Tawa P, Coppens M (2024). Acute rejection rates in vascularized composite allografts: a systematic review of case reports. J Surg Res.

[73] Schneeberger S, Lucchina S, Lanzetta M (2005). Cytomegalovirus-related complications in human hand transplantation. Transplantation.

[74] Duhamel P, Suberbielle C, Grimbert P (2015). Anti-HLA sensitization in extensively burned patients: extent, associated factors, and reduction in potential access to vascularized composite allotransplantation. Transpl Int.

[75] Jabłecki J, Kaczmarzyk L, Domanasiewicz A, Chełmoński A, Kaczmarzyk J (2010). Unsuccessful attempt of forearm transplantation--case report. Ann Transplant.

[76] Jabłecki J, Kaczmarzyk L, Domanasiewicz A, Chełmoński A, Kaczmarzyk J, Paruzel M (2010). Hand transplant - outcome after 6 months, preliminary report. Ortop Traumatol Rehabil.

[77] Jabłecki J, Syrko M, Arendarska-Maj A (2010). Patient rehabilitation following hand transplantation at forearm distal third level. Ortop Traumatol Rehabil.

[78] Jablecki J, Kaczmarzyk L, Domanasiewicz A, Chelmoński A, Kaczmarzyk J (2013). Unilateral hand transplant-results after 41 months. Transplant Proc.

[79] Cavadas PC, Thione A, Carballeira A, Blanes M (2013). Bilateral transfemoral lower extremity transplantation: result at 1 year. Am J Transplant.

[80] Barrow B, Diep GK, Berman ZP (2024). Immunologic outcomes in cross-sex solid organ transplants: a systematic review and meta-analysis to inform vascularized composite allotransplantation. Plast Reconstr Surg.

[81] Berry GJ, Burke MM, Andersen C (2013). The 2013 International Society for Heart and Lung Transplantation Working Formulation for the standardization of nomenclature in the pathologic diagnosis of antibody-mediated rejection in heart transplantation. J Heart Lung Transplant.

[82] Giarraputo A, Coutance G, Aubert O (2023). Banff human organ transplant consensus gene panel for the detection of antibody mediated rejection in heart allograft biopsies. Transpl Int.

[83] Kaufman CL, Cascalho M, Ozyurekoglu T (2019). The role of B cell immunity in VCA graft rejection and acceptance. Hum Immunol.

[84] Huelsboemer L, Moscarelli J, Dony A (2024). The role of C4d and donor specific antibodies in face and hand transplantation-a systematic review. Front Transplant.

[85] Huelsboemer L, Kauke-Navarro M, Boroumand S (2024). Ten-year follow-up after face transplantation-a single-center retrospective cohort study. Am J Transplant.

[86] Moktefi A, Hivelin M, Grimbert P (2021). Face transplantation: a longitudinal histological study focusing on chronic active and mucosal rejection in a series with long-term follow-up. Am J Transplant.

[87] Chaudhry A, Sosin M, Bojovic B, Christy MR, Drachenberg CB, Rodriguez ED (2015). Defining the role of skin and mucosal biopsy in facial allotransplantation: a 2-year review and analysis of histology. Plast Reconstr Surg.

[88] Ortona E, Pierdominici M, Rider V (2019). Editorial: sex hormones and gender differences in immune responses. Front Immunol.

[89] Klein SL, Flanagan KL (2016). Sex differences in immune responses. Nat Rev Immunol.

[90] Liu L, Karagoz H, Herneisey M (2020). Sex differences revealed in a mouse CFA inflammation model with macrophage targeted nanotheranostics. Theranostics.

[91] Jacobson DL, Gange SJ, Rose NR, Graham NM (1997). Epidemiology and estimated population burden of selected autoimmune diseases in the United States. Clin Immunol Immunopathol.

[92] Klein SL, Morgan R (2020). The impact of sex and gender on immunotherapy outcomes. Biol Sex Differ.

[93] Kumar R, Godavarthy PS, Krause DS (2018). The bone marrow microenvironment in health and disease at a glance. J Cell Sci.

[94] Yamada A, Arakaki R, Saito M, Tsunematsu T, Kudo Y, Ishimaru N (2016). Role of regulatory T cell in the pathogenesis of inflammatory bowel disease. World J Gastroenterol.

[95] Blumenstein I, Herrmann E, Filmann N (2011). Female patients suffering from inflammatory bowel diseases are treated less frequently with immunosuppressive medication and have a higher disease activity: a subgroup analysis of a large multi-centre, prospective, internet-based study. J Crohns Colitis.

[96] Velicković-Radovanović R, Mikov M, Paunović G, Djordjević V, Stojanović M, Cvetković T, Djordjević AC (2011). Gender differences in pharmacokinetics of tacrolimus and their clinical significance in kidney transplant recipients. Gend Med.

[97] Tan J, Wu W, Xu X (2012). Induction therapy with autologous mesenchymal stem cells in living-related kidney transplants: a randomized controlled trial. JAMA.

[98] Szajerka T, Klimczak A, Jablecki J (2011). Chimerism in hand transplantation. Ann Transplant.

[99] Wu S, Xu H, Ravindra K, Ildstad ST (2009). Composite tissue allotransplantation: past, present and future-the history and expanding applications of CTA as a new frontier in transplantation. Transplant Proc.

[100] Gorantla VS, Brandacher G, Schneeberger S, Zheng XX, Donnenberg AD, Losee JE, Lee WP (2011). Favoring the risk-benefit balance for upper extremity transplantation--the Pittsburgh Protocol. Hand Clin.

[101] Huelsboemer L, Stögner VA, Hosseini H (2024). Update on long-term mental health outcomes in eight face transplant recipients from a single center. Int J Psychiatry Med.

[102] Brandt L, Liu S, Heim C, Heinz A (2022). The effects of social isolation stress and discrimination on mental health. Transl Psychiatry.

[103] Coffman KL, Gordon C, Siemionow M (2010). Psychological outcomes with face transplantation: overview and case report. Curr Opin Organ Transplant.

[104] Grady KL, Naftel DC, White-Williams C (2005). Predictors of quality of life at 5 to 6 years after heart transplantation. J Heart Lung Transplant.

[105] O'Carroll RE, Couston M, Cossar J, Masterton G, Hayes PC (2003). Psychological outcome and quality of life following liver transplantation: a prospective, national, single-center study. Liver Transpl.

[106] Klapheke MM, Marcell C, Taliaferro G, Creamer B (2000). Psychiatric assessment of candidates for hand transplantation. Microsurgery.

[107] Murphy DC, Hoyle V, Saleh D, Rees J, Bound Alberti F (2021). Central importance of emotional and quality-of-life outcomes in the public's perception of face transplantation. Br J Surg.

[108] Vanterpool KB, Gacki-Smith J, Downey MC (2023). Patient preferences of patient selection criteria for upper extremity vascularized composite allotransplantation: a qualitative study. SAGE Open Med.

[109] Cavaliere A, Rega U, Grimaldi S (2024). Long-term outcomes and future challenges in face transplantation. J Plast Reconstr Aesthet Surg.

[110] Fischer S, Kueckelhaus M, Pauzenberger R, Bueno EM, Pomahac B (2015). Functional outcomes of face transplantation. Am J Transplant.

[111] Lanzetta M, Petruzzo P (2007). A comprehensive functional score system in hand transplantation. Hand Transplantation.

[112] Wollesen B, Gräf J, Hansen L, Gurevich A, Elprama SA, Argubi-Wollesen A, De Pauw K (2024). Gender differences in the use of an upper-extremity exoskeleton during physically and cognitively demanding tasks - a study protocol for a randomized experimental trial. Front Neurol.

[113] Tanaka K, Fujita N, Higashi Y, Ogawa N (2002). Neuroprotective and antioxidant properties of FKBP-binding immunophilin ligands are independent on the FKBP12 pathway in human cells. Neurosci Lett.

[114] Kay SP, Leonard DA (2023). Hand transplantation: can we balance the risks and benefits?. J Hand Surg Eur Vol.

[115] Tuffaha SH, Budihardjo JD, Sarhane KA (2016). Growth hormone therapy accelerates axonal regeneration, promotes motor reinnervation, and reduces muscle atrophy following peripheral nerve injury. Plast Reconstr Surg.

[116] Wideman L, Weltman JY, Shah N, Story S, Veldhuis JD, Weltman A (1999). Effects of gender on exercise-induced growth hormone release. J Appl Physiol (1985).

[117] Rath J, Zhou X, Lee EB (2024). The effects of growth hormone on nerve regeneration and alloimmunity in vascularized composite allotransplantation. Plast Reconstr Surg.

[118] Faroni A, Mobasseri SA, Kingham PJ, Reid AJ (2015). Peripheral nerve regeneration: experimental strategies and future perspectives. Adv Drug Deliv Rev.

